# Promoting physical activity and physical functioning among residents in nursing homes – lessons learnt by the PROGRESS project

**DOI:** 10.3389/fragi.2026.1793208

**Published:** 2026-08-03

**Authors:** Vera Belkin, Berit K. Labott, Claudia Voelcker-Rehage

**Affiliations:** 1 Department of Neuromotor Behavior and Exercise, Institute of Sport and Exercise Sciences, University of Münster, Münster, Germany; 2 JICE, Joint Institute for Individualisation in a Changing Environment, University of Münster and Bielefeld University, Münster, Germany

**Keywords:** environmental intervention, exercise intervention, health promotion, healthy ageing, inpatient care facility, organizational readiness, primary prevention

## Abstract

**Introduction:**

Nursing home residents show high sedentary behavior and limited physical functioning, while structural constraints hinder sustainable prevention. This study examined how group-based physical exercise and environmental interventions, such as activity-promoting materials and interior design, can be integrated into everyday care to promote a physical activity–promoting culture in nursing homes and identified key organizational and individual determinants of sustainable implementation.

**Methods:**

PROGRESS was a participatory, cluster-randomized cross-over trial conducted in seven nursing homes in the Münster area in Germany. Residents received two of four guided or non-guided exercise (n = 62) and/or environmental interventions (e.g., activity posters, a seated bike ergometer, walking brochures/routes; n = 65). Data from attendance logs, participatory workshops, and a follow-up questionnaire at t4 (the fourth and final measurement time point, approximately 50 weeks after baseline assessment; n = 73) were analyzed using descriptive statistics and structured content analysis for open-ended feedback. Based on these findings, the PROGRESS pyramid was developed as a framework for sustainable implementation of physical activity into everyday care.

**Results:**

Of the 73 participants, 90.4% attended at least one guided intervention session. Participation was highest in the exercise intervention, with most residents reporting weekly engagement. In contrast, engagement with environmental interventions was more variable and strongly dependent on staff support, with 63.0% of residents reporting no independent use of physical activity opportunities. Comparisons between self-reported participation and attendance records revealed substantial discrepancies, particularly for environmental interventions. Overall satisfaction with the project was high (mean = 2.7 ± 2.5 on a 0 = happy to 10 = angry scale). Most participants supported continuation of the interventions (86.0%), and 64.0% reported perceived personal benefits. Organizational and individual determinants jointly shaped participation.

**Conclusion:**

In order to promote physical activity and functioning sustainably in nursing homes, it is necessary to address organizational conditions and individual needs in a coordinated manner. The PROGRESS pyramid translates these findings into a practical framework comprising organizational and communicational foundations, resident participation, education, and staff qualification to guide the long-term integration of exercise and environmental interventions into daily routine.

## 1 Introduction

Like many Western countries, Germany is facing significant demographic changes that are putting pressure on its healthcare and long-term care systems (ISSA, 2022; Koutsogeorgopoulou and Morgavi, 2025). Population ageing is accompanied by a rising prevalence of chronic conditions and care dependency, resulting in substantial demands on social security systems and driving up health costs (Bujard and Dreschmitt, 2016). While the majority of individuals in need of care in Germany (approximately 80%) receive assistance in their own homes, long-term care facilities are facing increasing structural challenges. Nursing homes are affected by persistent staff shortages, which limit their capacity to meet residents’ care needs. Research indicates that staffing levels in German nursing homes, similar to those in many other Western countries, remain insufficient to ensure adequate care provision (Alber, 1990; Maier and Afentakis, 2013; Heger et al., 2025; Miller et al., 2025). Current estimates suggest that a 36% increase in nursing staff would be necessary to maintain acceptable care quality standards under current conditions (Maier and Afentakis, 2013; Burfeindt et al., 2024; Heger et al., 2025).

These structural constraints have direct consequences for everyday life in nursing homes. Nursing home residents typically spend around 80% of their waking hours sitting or lying down, exhibiting only minimal physical activity (Rezende et al., 2014; Auerswald et al., 2020). They are therefore more sedentary than residents in assisted living facilities, with the most common sedentary activities being watching television and taking short naps (Marmeleira et al., 2017). Studies have found that residents take an average of 863 to 1.678 steps per day, far below recommended levels for maintaining functional capacity (Rezende et al., 2014; Auerswald et al., 2020). In addition to individual preferences and complaints, residents’ physical activity is significantly influenced by organizational structures, staffing levels, institutional routines, and the physical environments that discourage movement (Douma et al., 2017; Jansen et al., 2017; Hawkins et al., 2018). Physical inactivity is associated with limited mobility within the living space, which describes the extent to which individuals move within and outside their immediate living environment (Tsai et al., 2015).

As a consequence, physical inactivity in nursing homes often develops into a self-reinforcing cycle: it is associated with reduced physical and cognitive performance, limited independence, and lower quality of life, which in turn further restricts mobility and participation in activities of daily living. Limited mobility within the living environment is also influenced by age-related cognitive deficits, particularly with regard to spatial navigation and orientation (Moffat et al., 2001; Barnes et al., 2007; Tsai et al., 2015; De Silva et al., 2019). These deficits can restrict mobility in both familiar and unfamiliar surroundings. This highlights the need for structured, system-level prevention strategies in long-term care.

In response to the growing burden of disease caused by demographic changes, chronic diseases, and social inequality in the health sector, Germany enacted the Act to Strengthen Health Promotion and Prevention (Prevention Act) in July 2015. The act aims to improve cooperation between social insurance providers, federal states, and local authorities in prevention and health promotion. As part of this legislation, long-term care insurance providers were given a specific prevention mandate to provide health promotion services to people in nursing homes (GKV-Spitzenverband, 2023). The National Association of Statutory Health Insurance Funds (in German: GKV-Spitzenverband) subsequently developed a prevention guideline that covers five areas of action: nutrition, physical activity, cognitive resources, psychosocial health, and prevention of violence. The present article focuses on interconnected areas of physical activity, cognitive resources, and psychosocial health (GKV-Spitzenverband, 2024). Further, the guidelines recommend a setting- or living environment-based approach for health promotion in nursing homes, combining exercise (group-based physical exercise program) with environmental interventions. The rationale is that health-promoting conditions facilitate motivation, learning, and practice of health-promoting behavior. Additionally, this approach aims to encourage self-determined, health-oriented behavior to prevent or reduce the risk of illness. The guidelines explicitly state that the target groups and stakeholders, which include nursing home residents, nursing and social care staff, facility management, and external parties such as therapists, doctors and family members, comprise all individuals involved in or affected by the health promotion process. This refers directly to achieve greater acceptance and success rates, they should be actively involved throughout the entire health promotion process through a participatory approach. Furthermore, a needs assessment is required to adapt measures to individual circumstances and needs (GKV-Spitzenverband, 2023; GKV-Spitzenverband, 2024).

Evidence-based measures to increase physical activity and cognitive functioning have preventive potential, as they strengthen health resources and counteract age-related functional decline (Weening-Dijksterhuis et al., 2011; Brett et al., 2016). However, existing research reveals a critical implementation gap. Studies have predominantly focused on either exercise interventions (Wollesen and Voelcker-Rehage, 2014; Cordes et al., 2019) or on creating activity-promoting environments (Fleiner et al., 2015; Levinger et al., 2023). While exercise interventions focus on improving individual functioning (Dechamps et al., 2010; Arrieta et al., 2018; Barrett et al., 2021), environmental interventions aim to create an environment conducive to physical activity and change behavior (Anderiesen et al., 2014; Douma et al., 2017). While both approaches show positive results, they differ in their specific motor, cognitive, and psychosocial goals and effects. Classic exercise interventions, such as strength training (Fiatarone et al., 1994; Weening-Dijksterhuis et al., 2011), balance training (e.g., tai chi or yoga) (Lazowski et al., 1999; Lach and Parsons, 2013; Cordes et al., 2019), moderate endurance training (Sauvage et al., 1992; Arrieta et al., 2018), or a combination of these elements (Wollesen and Voelcker-Rehage, 2014; Cordes et al., 2019; Wollesen et al., 2020), address physical functioning. Physical functioning refers to an individual’s physical capacities, such as strength, balance, mobility, and the ability to perform essential motor tasks (Caspersen et al., 1985). In contrast, environmental interventions, aim to increase physical activity levels and mobility within the living environment. These approaches comprise the integration of exercise routines into everyday life (Braun et al., 2016; Cordes et al., 2021), the redesign of the physical environment to encourage movement through specific, low-threshold offerings, such as activity posters or indoor and outdoor walking brochures. These materials are designed specifically to encourage spontaneous movement in residents’ everyday living spaces (Baandrup and Jennum, 2021; Bourdon and Belmin, 2021), and the creation of group activity rooms (Fleiner et al., 2015; Mouton et al., 2017; Levinger et al., 2023). Physical activity refers to any movement that increases energy expenditure, including activities of daily living, exercise, and recreational movement (Bull et al., 2020), and is typically reported as the number of steps or active minutes per day. Therewith, physical activity refers to the movement behavior itself, whereas physical functioning represents physiological capabilities.

To effectively promote physical activity level and functional capacity among nursing home residents, both intervention types must be combined: the entire living environment needs to be designed to encourage movement through specific offerings like physical activity opportunities, complemented by exercise programs that promote specific physical functions such as balance, strength, or walking speed. To date, only a few programs for nursing home residents meet this integrated approach.

Despite the strong evidence for both exercise and environmental interventions, they remain underutilized in daily routine. Structural barriers often hinder the successful and sustainable implementation of these prevention approaches in everyday care (Otto et al., 2025). Central challenges include time constraints, staff shortages, and insufficient financial resources (Roßbach et al., 2026). Shift and staff changes, combined with inadequate communication structures, for example, result in information losses (Köpke et al., 2013; Wollesen et al., 2023). The unpredictable nature of everyday life in nursing homes further complicates the integration of structured services. Additional obstacles include residents’ scheduling commitments and their varying physical and cognitive conditions (Wollesen et al., 2023). At the organizational level, unclear processes and a lack of strategic anchoring negatively impact participation rates and feasibility (Wollesen et al., 2023). Smooth integration of prevention concepts requires operational management and evaluation structures (Marent et al., 2014; Purwins and Roes, 2022; Schaller et al., 2024), as well as clear strategic leadership and organizational support from management with defined decision-making structures (Köpke et al., 2013; Huber, 2024).

This article presents PROGRESS, a comprehensive model project developed together with an insurance company in Germany that addresses these gaps. PROGRESS combines group-based physical exercise interventions with activity-promoting environments to improve physical functioning, increase physical activity level, and expand mobility within the living environment (Belkin et al., 2025). The goal was to establish a culture of physical activity anchored in nursing homes’ mission statements. Here, we addressed two primary questions: (1) How can exercise and environmental interventions be effectively integrated to create a sustainable culture of physical activity in nursing homes? (2) What organizational and individual factors determine the successful implementation and long-term continuation of such programs? By answering these questions, this article provides evidence-based guidance for implementing sustainable physical activity promotion in nursing home settings. This contributes to both practical application and theoretical understanding of complex health interventions in nursing homes.

## 2 Methods

### 2.1 Study design and approach

PROGRESS is a participatory, monocentric, cluster-randomized, controlled crossover study with seven intervention sites conducted between August 2022 and February 2025. Seven participating nursing homes were randomly allocated to two of four intervention groups before the baseline assessment (t1). Residents completed a battery of standardized physical, cognitive, and psychosocial assessments at four time points spaced 16 weeks apart (t1–t4). In addition to the standardized test battery, follow-up questions were collected through brief structured interviews at t4. The first intervention phase occurred between t1 and t2, the second phase occurred between t2 and t3, and the follow-up phase occurred between t3 and t4. PROGRESS primarily aimed to evaluate the effectiveness of the four interventions on residents’ physical functioning and activity levels (Belkin et al., 2025). The current sub-analysis examined implementation processes and sustainability factors within PROGRESS. Therefore, participation patterns, stakeholder feedback, as well as organizational conditions were analyzed, and a practical framework for sustainable physical activity promotion in nursing homes was developed.

### 2.2 Participants characteristics

A total of 123 participants from seven nursing homes in the Münster area, Germany, were enrolled at baseline. Of these, 80 participants completed all the study assessments up to the follow-up measurement (t1-t4). Seven of them were unable to answer the follow-up questionnaire for cognitive reasons. This resulted in an analytical sample of *n = 73* for the current study. Participants’ baseline characteristics are summarized in Table 1. Eligibility criteria included voluntary participation, a minimum age of 60 years, the ability to participate in group training and follow instructions, and the capacity to sit independently in a wheelchair. Participants who no longer met these criteria due to age-related functional or cognitive decline were excluded from the study but were permitted to continue engaging with the interventions voluntarily outside of the study analyses (Belkin et al., 2025). The study was conducted in accordance with the Declaration of Helsinki and Good Clinical Practice guidelines. Ethics approval was granted by the Ethics Committee of the Faculty of Psychology and Sports Science at the University of Münster, Germany (protocol 2022-40-CVR), and the study was registered at DRKS. de (registration number DRKS00031020, 23.02.2023).

**TABLE 1:** Participants’ baseline characteristics.

Characteristics	Participants N = 73
Age [years]	83.92 (8.05)
Sex [m/f]	17/56
Height [m]	1.62 (0.11)
Weight [kg]	70.45 (15.41)
BMI [kg/m2]	26.86 (4.76)
MoCA [points]	15.71 (5.16)
N diagnoses	11.45 (7.15)
N medication	10.83 (4.78)
Walking aid [N without/N rollator/N wheelchair]	18/45/10
Walking ability [yes/no]	63/10
N participants who fell	​
Only self-reported	26
Only nurse reported	11
Both reporting	6
Education [>12 years/≤12 years]	20/53

### 2.3 Participatory approach

The PROGRESS study employed a participatory approach to ensure that the interventions were contextually appropriate, acceptable, and aligned with the needs of residents and staff. Therefore, in each nursing home, a steering committee was established as an institutional decision-making structure. Across the seven nursing homes, steering committees typically comprised three to eight members, including at least one representative from facility management, one from nursing or social care staff, and one resident representative where cognitive capacity permitted. The exact composition was tailored to each facility’s organizational structure and available personnel. Due to the COVID-19 pandemic at the start of the project, it was not possible to include residents of nursing homes in the steering group at all facilities (see Table 2).

**TABLE 2:** Compositions of the steering committees in the PROGRESS project.

Nursing home	Head of NH	Social service	Nursing staff	Residents	Total number
NH1	1	​	1	4	6
NH2	​	3	​	​	3
NH3	1	2	​	5	8
NH4	1	​	2	3	5
NH5	1	1	​	3	5
NH6	​	1	​	6	7
NH7	1	2	​	5	8

NH, nursing home. Steering committee composition varied across facilities to reflect each nursing home’s organizational structure and available personnel. Total numbers reflect the overall number of individuals contributed by each facility, as multiple representatives from one group were possible (e.g., NH2: three social service staff; NH7: one head of nursing home, two social service staff, five residents).

Its role was to discuss organizational conditions, human resources, environmental constraints, and residents’ needs, and to guide the participatory development and implementation of physical activity-promoting measures.

As part of the participatory approach, the steering group conducted two needs assessments in each nursing home: the first 1 month before the baseline assessments, as a kick-off workshop (t1), and the second between t3 and t4, to evaluate and refine the intervention components. The aim was to ensure that both the exercise and environmental interventions reflected the context-specific needs and capabilities of each nursing home and supported sustainable integration into everyday routines. The needs assessment followed a structured, multi-step process based on an established participatory framework (Wollesen et al., 2023; GKV-Spitzenverband, 2024; Belkin et al., 2025).

1. Assessment of the current situation:

The current state of physical activity promotion in the nursing home was evaluated, and the desired future state was collaboratively defined. This included an assessment of how residents used different living spaces, guided by the Life Space Mobility concept (Jansen et al., 2017).

2. Identification of barriers and resources:

Physical, cognitive, organizational, and architectural barriers were systematically recorded, together with existing human, spatial, and material resources relevant for facilitating physical activity.

3. Development of action strategies:

Based on the identified needs, stakeholders jointly generated potential physical exercise and environmental intervention strategies. Feasibility, acceptability, and priority were assessed collaboratively, and both short- and long-term goals were defined.

4. Implementation planning:

Specific measures were selected for implementation within 4 to 6 weeks, and responsibilities and timelines were established. The repeated needs assessment served not only to adapt interventions to evolving resident and organizational needs but also to evaluate existing measures and replace those that had not been used or effective.

### 2.4 Interventions

All intervention components were developed based on the results of the participatory needs assessment and tailored to the context of each nursing home. The intervention package consisted of two core elements: a group-based physical exercise program and an environmental intervention designed to increase physical activity opportunities throughout the living environment (for further details, cf. (Belkin et al., 2025)).

#### 2.4.1 Exercise intervention

The group-based physical exercise intervention program comprised a structured group program (32 sessions of 45–60 min, twice weekly) in accordance with the International Association of Gerontology and Geriatrics guidelines. This program was based on two preceding studies, PROCARE (Cordes et al., 2019) and PROfit (Wollesen et al., 2020), and was led by trained exercise and sport scientists or physical therapists. The content included warm-ups, exercises to improve coordination, spatial orientation, and cognition, as well as strength training, gait training, and cool-downs. All components followed progressive increases and individual adjustments.

#### 2.4.2 Guided and non-guided environmental interventions

The environmental intervention programs involved installing permanent physical activity opportunities aiming to promote physical activity throughout the residents’ living spaces. To this end, it adopted the Life Space Mobility concept, which categorizes the living spaces of nursing home residents into four zones: their own room (zone 1), the ward corridor (zone 2), the area outside their ward (zone 3), and the immediate surroundings of the building (zone 4) (Jansen et al., 2017). While physical activity opportunities were offered in zones one to three of the living space (including activity posters, darts, ball games, indoor walking brochures, and seated bike ergometer), outdoor routes (“rollator club”) were provided for zone 4. In the guided version, 45–60 min guided sessions took place twice a week, whereas in the non-guided version, after the physical activity opportunities were set up, the nursing home staff were responsible for integrating them into everyday life. The physical activity opportunities were installed permanently, so that the nursing home residents could also use them outside the guided slots. During the follow-up phase, all groups received a non-guided environmental intervention.

#### 2.4.3 Physical activity-promoting culture

Together, the exercise and the guided environmental intervention formed a comprehensive, physical activity-promoting culture. It offers four supervised (45–60 min) sessions per week (64 in total). The permanent presence of environmental opportunities to be physically active, combined with the structured exercise sessions, was intended to encourage both planned and spontaneous physical activity, support habit formation, and create a sustainable foundation for active living within the institutional context.

### 2.5 Data collection procedure

In the PROGRESS study, various physical activity, physical functioning, psychological, and cognitive parameters were collected (Belkin et al., 2025). In this manuscript, however, our focus is exclusively on the qualitative results from the workshops and the follow-up interviews.

#### 2.5.1 Kick-off workshop

The kick-off workshop (conducted 1 month before t1) served to assess current physical activity practices and to collaboratively develop intervention strategies following the participatory approach as described in Section 2.3. Data collected included documentation of identified barriers, resources, and prioritized intervention strategies.

#### 2.5.2 Intervention participation

Participation in all guided intervention sessions, comprising the exercise sessions and the guided environmental activity opportunities, was documented by trained instructors using standardized attendance protocols.

#### 2.5.3 Final workshop

Between t3 and t4, the second participatory workshop was conducted in each nursing home with all steering committee members. The workshops served to evaluate the implementation of both intervention components, to assess the extent to which goals from the initial needs analysis had been achieved, and to identify remaining barriers and facilitators. Each workshop involved a short presentation summarizing the intervention activities since t1, followed by structured group discussions. Notes and written feedback were collected by the research team.

#### 2.5.4 Follow-up questionnaire

After the follow-up phase (t4), participants were invited to complete a self-administered questionnaire assessing their experiences with the interventions. These eighteen questions were organized into three response categories and contained qualitative as well as quantitative questions. The questionnaire was administered as a structured interview by trained research staff.

##### 2.5.4.1 Self-reported satisfaction

Participants reported their overall satisfaction with the project, the provided clarification and process information, and the long-term use of a pedometer (Fitbit Zip, used for activity monitoring in the main study). In addition, satisfaction with physical activity opportunities offered by the environmental interventions was evaluated. Responses were assessed by using a symbolic visual analog scale (VAS) with a movable slider presenting six smiley faces ranging from happy to angry. Each participant operated the slider manually. The numerical value on the reverse of the slider, ranging from 0 (happy smiley) to 10 (angry smiley), was used for quantitative analysis.

##### 2.5.4.2 Self-reported participation

Self-reported participation was assessed by five items on a seven-point Likert scale (daily - once a week - several times a week - once a month, several times a month, less often - never). Participants were asked to self-report on their participation in the exercise intervention and physical activity opportunities. Participants who selected ‘never’ were asked to provide a brief, verbal, open-ended explanation for their non-participation. This explanation was transcribed by the interviewer.

##### 2.5.4.3 Self-reported motivation and interest

Participants were asked to respond to six yes-or-no questions about using the interventions outside of the guided, fixed time slots, either alone, with their family, or with the nursing home staff. We also asked them whether they felt the interventions had been beneficial, and if they saw any added value in the services offered. Furthermore, the residents were asked whether they would like to continue receiving the interventions, and why. The verbal, open-ended argumentations were written down by the interviewer.

### 2.6 Data analysis

Quantitative and qualitative data were analyzed using a mixed-methods approach. All analyses presented in this article refer to the subsample of n = 73 participants who completed the follow-up assessment and had sufficient cognitive capacity to provide feedback. Based on these findings, the PROGRESS pyramid was developed to synthesize key implementation factors into a practical model for the sustainable implementation of a physical activity-promoting culture.

#### 2.6.1 Quantitative analysis

Descriptive statistics (means, standard deviations, ranges, frequencies, and percentages) were calculated for intervention attendance, satisfaction ratings, and self-reported participation. Based on the distribution of responses, the seven-point Likert scale for participation was collapsed into three categories: “weekly” (daily, once a week, several times a week), “monthly” (once a month, several times a month, less often), and “never” (unchanged). Documented participation from attendance logs was compared with self-reported participation to assess recall accuracy through categorical agreement analysis. Responses were coded as “consistent” or “inconsistent” using conditional logic (IF function) to identify matches between documented and self-reported attendance categories. The analysis was performed using IBM SPSS Statistics (version 31.0.0.0 (117)).

#### 2.6.2 Qualitative analysis

The qualitative material consisted of short open-ended comments linked to specific questionnaire items (e.g., explanations for non-participation) as well as notes from the participatory workshops. Given the brevity of these responses, the analytical focus was on identifying recurring categories rather than conducting in-depth interpretive analysis. The data were examined using structured content analysis to categorize frequently mentioned barriers, facilitators, and explanatory themes with MAXQDA (VERBI Software, version 24.1.0). Coding was conducted by two researchers independently, and discrepancies were resolved through discussion until consensus was reached. Themes were subsequently organized into higher-order categories representing organizational and individual determinants of implementation.

## 3 Results

### 3.1 Participants flow

**FIGURE 1:**
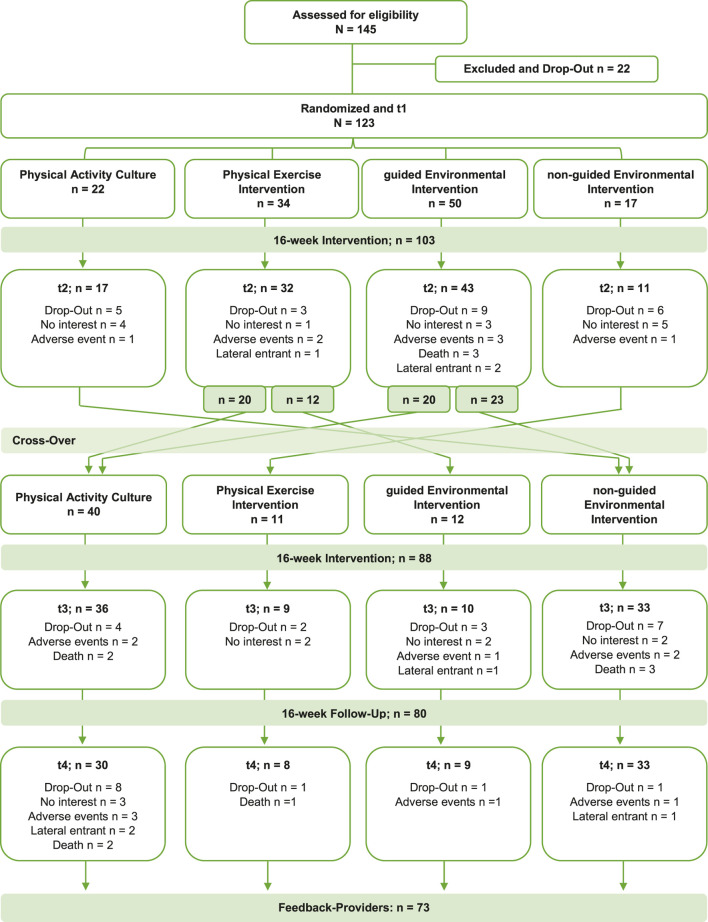
Participant flow. *Note:* The diagram illustrates the flow of participants across the four intervention groups and the two active intervention phases, which are separated by a crossover at t2. Group sizes after the crossover reflect reallocation according to the original randomization scheme. As a result, out of the final feedback-providers sample of 73 participants, 62 participated in exercise intervention and 65 in environmental intervention.

Of the 123 participants initially enrolled, 80 completed all assessments through t4. In total, 50 participants withdrew during the study: 22 due to lack of interest or unwillingness to change established routines, 17 due to adverse events (falls, disease exacerbations, cognitive decline), and 11 deaths. Additionally, seven new participants entered the study after baseline. As seven participants who completed t4 could not complete the follow-up questionnaire due to cognitive limitations, the final analytical sample comprised 73 participants (see Figure 1). All newly enrolled participants were allocated according to the original crossover scheme.

During the first intervention phase (t1–t2), 23 participants withdrew (13 lack of interest, 7 adverse events, 3 deaths), while 3 new participants enrolled after baseline. During the second phase (t2–t3), 16 dropped out (6 lack of interest, 5 adverse events, 5 death) while one new one joined in. During the follow-up phase (t3-t4), 11 dropped out (3 lack of interest, 5 adverse events, 3 death) while three were newly enrolled after t3.

### 3.2 RQ1 - Integration of exercise and environmental interventions

#### 3.2.1 Intervention participation, self-reported participation and recall accuracy

Based on the crossover design (T1-T2 and T2-T3), 62 out of 73 participants were assigned to an intervention arm that included a physical exercise program (physical activity–promoting culture or exercise intervention), and 65 were assigned to an intervention arm that provided guided environmental adaptations (physical activity–promoting culture or guided environmental intervention). Documentation shows that 82.3% (n = 51) of the 62 residents participated in the exercise intervention. Regarding the guided environmental intervention, the documentation showed that 75.4% (n = 49) of the 65 residents indeed participated. Overall, out of the 73 participants, 90.4% (n = 66) attended at least one guided intervention session. Of those who participated in the exercise intervention, 79.0% (n = 49) attended more than one session (21.9 ± 17.7 sessions (range: 0.0–58.0). Similarly, of those who participated in the guided environmental intervention, 58.5% (n = 38) attended more than one session (8.9 ± 10.9 sessions (range: 0.0–54.0)).

##### 3.2.1.1 Exercise intervention

Among the 62 participants who received the exercise intervention, recall accuracy was 79.0% (n = 49), with consistent agreement between documented attendance and self-reported participation. Of those with consistent recall, 85.7% (n = 42) reported weekly participation, and 14.3% (n = 7) reported never participating.

Of the participants with inconsistent recall (n = 13), the majority (n = 7, 53.8%) had documented participation but reported either no memory of the intervention (n = 4) or never having participated (n = 3). Six participants had no documented participation but reported either monthly attendance (n = 1) or no memory of the intervention (n = 5).

##### 3.2.1.2 Guided environmental intervention

Among the 65 participants who received the guided environmental intervention, recall accuracy over all physical activity opportunities was 56.9% (n = 37), with consistent agreement between documented attendance and self-reported participation. Reporting varied considerably across the different physical activity opportunities. Participation in activity posters (n = 23, 50.0%) and the seated bike (n = 40, 61.5%) were recalled more accurately than participation in darts (n = 9, 30.0%), ball games (n = 12, 40.0%), the rollator club (n = 29, 44.6%), and indoor walking brochures (n = 16, 45.7%).

Inconsistent recall rates ranged from 70.0% (n = 21) for darts, over 60.0% (n = 18) for ball games, 55.4% (n = 36) for the rollator club, 54.3% (n = 19) for indoor walking brochures, 50.0% (n = 23) for activity posters, to 38.5% (n = 25) for the seated bike. The main reason for inconsistent recall at all physical activity opportunities was that participants took part in guided environmental sessions, but then recalled not taking part or forgetting about their attendance. A minority of participants answered that they used the physical activity stations weekly or monthly without documented participation by a trainer (activity posters n = 5, 10.9%; seated bike n = 10, 15.4%; darts n = 0; ball games n = 1, 3.3%; rollator club n = 4, 6.2%; indoor-walking brochure n = 3, 8.6%). Detailed information about consistent and inconsistent recall accuracy is presented in Supplementary Table S1.

##### 3.2.1.3 Non-guided environmental intervention

Unfortunately, the nursing home staff did not record participation in the non-guided environmental intervention. Therefore, the evaluation is only based on the number of participants in the guided environmental intervention.

The independent self-reported use of physical activity opportunities outside of guided sessions was limited. In total, 63.0% of participants (n = 73) reported never engaging in physical activity opportunities independently, with relatives, or with staff. Of those who used the physical activity opportunities outside of guided sessions, 23.3% initiated use independently, while 19.2% only participated with staff assistance. Use of physical activity opportunities with family members was uncommon (5.5%).

#### 3.2.2 Self-reported satisfaction

Overall satisfaction with the project and its components was good. Participants (n = 73) rated the intervention with a mean score of 2.7 ± 2.5 on the visual analogue scale from 0 (happy smiley) to 10 (angry smiley), and the information provided during the study received a rating of 2.7 ± 2.3. The physical activity opportunities were also rated as good, with 3.0 ± 1.8. Most participants expressed support for continuing the interventions: 86.0% said they would like the activities to continue, and 64.0% reported personal benefits.

#### 3.2.3 Use and usability of pedometers

Regarding the use of pedometers, 26.0% (n = 19) of participants raised concerns about their usability, even though they had decided against wearing them from the beginning. Although pedometers were provided to nursing staff for role modelling purposes, 85.1% of residents (n = 39) reported not noticing staff wearing them, and only 5.6% (n = 3) found them motivating. Among residents who wore a pedometer, 95.7% (n = 44) reported that the wearing time was appropriate, and 4.3% (n = 2) felt the wearing time was too long.

#### 3.2.4 Kick-off and final workshop: implementation and culture-building indicators

At the kick-off workshop, both residents and staff commonly expressed concerns about the intensity of the planned intervention schedule. The originally intended four supervised sessions per week were considered potentially overwhelming, especially for residents with limited energy or other daily commitments. During the final workshops, participants frequently advocated reducing the frequency to one guided session per week for each intervention type (exercise + environmental).

### 3.3 RQ2 - Determinants of sustainable program implementation

#### 3.3.1 Organizational factors

Feedback from workshops and follow-up interviews identified organizational readiness and resource allocation as critical factors for successful implementation. Social service employees demonstrated a high level of willingness and motivation to implement group-based physical exercise programs. They expressed satisfaction with the support they received from the researchers and appreciation for the new ideas to promote physical activity. Staff particularly valued environmental prevention measures as “low-threshold” tools that could easily be incorporated into routine care activities. However, initial skepticism about the proposed frequency of four sessions per week reflected practical concerns about resource allocation and resident engagement capacity.

Several structural requirements were essential for successful implementation. Feedback from workshops and follow-up interviews consistently identified systematic guidance and reminder systems as vital components for encouraging residents’ participation.

#### 3.3.2 Individual factors: self-reported motivation and interest

Analysis of open-ended responses about self-reported motivation and interest revealed distinct patterns of participation barriers across the different components of the intervention. Residents who stated that they had neither participated in the intervention nor used the physical activity opportunities were asked to provide an open-ended response. The following results present the residents’ answers, regardless of whether they corresponded with our documentation of their participation in the intervention. Furthermore, intrinsic and extrinsic motivational factors could be identified, and both influenced participation. Tangible rewards such as certificates and stamp cards were frequently mentioned as helpful extrinsic motivators during the follow-up workshop. Intrinsic motivation was reflected in 64.0% of participants (n = 47/73) who reported personal benefit from the interventions. Satisfaction with specific components, such as pedometer use, further reflected how design features supported or hindered motivation; 95.7% of pedometer users considered the wearing time acceptable.

The availability of multiple intervention formats (group-based physical exercise, physical activity opportunities, guided and non-guided sessions) aligned with the diverse preferences and functional capacities of the resident population.

##### 3.3.2.1 Exercise intervention

Among the participants assigned to the exercise intervention (n = 62), 16.1% (n = 10) reported that they had never participated. The primary barriers were limited awareness of session availability (n = 5), temporary health symptoms like dizziness (n = 1), perceived redundancy with existing physiotherapy services (n = 1), and psychosocial factors, including aversion to group-based activities or a preference for solitary engagement (n = 3).

##### 3.3.2.2 Guided environmental intervention

Staff support notably influenced engagement in physical activity opportunities. Documented attendance showed that 19.2% of participants (n = 14 out of 73) used physical activity opportunities outside guided sessions when accompanied by staff. In contrast, no independent use occurred among those without staff support.

Participants who did not engage with the physical activity opportunities outside the guided sessions identified several barriers to participation. These barriers were categorized into four main themes: lack of awareness, motivational factors, need for accompaniment or assistance, and individual preferences or constraints.

A lack of awareness was identified as the most frequently reported barrier (n = 43) to all physical activity opportunities. This was particularly evident in relation to the seated bike, with 16 participants reporting that they were either unaware of the opportunity or had not been invited to participate. Conversely, these arguments were raised only twice in relation to the ball games.

Motivational barriers were the second most frequently mentioned barrier (n = 30). Some arguments related to multiple physical activity opportunities, such as low interest or lack of motivation (n = 13), and feeling “too lazy” (n = 10). Other arguments were specific to one physical activity opportunity, such as not engaging when passing by (n = 2, activity posters), perceiving the activity as unhelpful (n = 1, activity posters), or explicitly expressing a lack of interest in the activity itself (n = 4, darts).

Individual preferences and practical constraints emerged as diverse and personalized barriers to participation (n = 25). Some residents preferred to follow their personal routines (n = 10) or use their own equipment, such as keeping their own seated bike in their room (n = 3). Others named limited time or competing commitments (n = 3), hospital stays (n = 1), “Walked enough in life” (n = 4), not having thought about joining (n = 2), not seeing the point (n = 1), or felt it was enough to know that he/she could still perform the exercises (n = 1).

Finally, the need for accompaniment or assistance (n = 15) was identified as a barrier primarily relating to functional limitations and safety concerns. This was specifically mentioned for the indoor walking brochures (n = 4) and the rollator club (n = 6). Participants reported a lack of willingness to exercise alone, functional limitations requiring assistance (e.g., wheelchair use, walking difficulties, visual impairments), safety concerns, and not having received the necessary accompaniment. Supplementary Table S2 provides detailed information about the barriers to independent participation in the different physical activity opportunities.

## 4 Discussion and reflection

This study aimed to examine how exercise and environmental interventions can be integrated to promote a sustainable culture of physical activity in nursing homes (RQ1) and to identify the organizational and individual determinants influencing their long-term implementation (RQ2). The findings showed that the group-based physical exercise program was well integrated into daily routines and achieved consistently high participation rates, whereas engagement with the environmental interventions was considerably more variable and strongly dependent on staff support. The mean number of attended sessions further highlights this disparity: participants attended an average of 21.9 ± 17.7 (range: 0–58) exercise sessions and 8.9 ± 10.9 (range: 0–54) guided environmental sessions. These wide ranges and high variances indicate substantial heterogeneity in participation across residents and facilities: some residents attended continuously throughout the intervention period, while others participated rarely or not at all. This highlights that participation rates alone, such as the 90.4% of residents attending at least one guided intervention session, and 63.0% never used physical activity opportunities independently, may mask considerable variation in the actual dose of intervention received and should therefore be interpreted with caution.

Attrition patterns and self-reported reasons for non-participation highlighted a combination of awareness-related issues, health limitations, and individual preferences as key barriers at the personal level. At the organizational level, staff motivation to deliver the interventions was generally high, but feasibility concerns, particularly regarding intervention frequency, communication flow, and available resources, restricted consistent implementation across facilities. Together, these findings reveal two interconnected clusters of determinants that shaped implementation outcomes: organizational structures and processes and individual-level factors such as functional capacity, preferences, and motivational orientations. Both clusters influenced whether components were noticed, accessed, and sustained over time, suggesting that neither organizational readiness nor individual motivation alone is sufficient for successful integration. Instead, their interplay determines whether interventions become embedded within everyday routines and can contribute to the development of a facility-wide physical activity–promoting culture.

### 4.1 Building blocks of promoting sustainable physical activity

**FIGURE 2:**
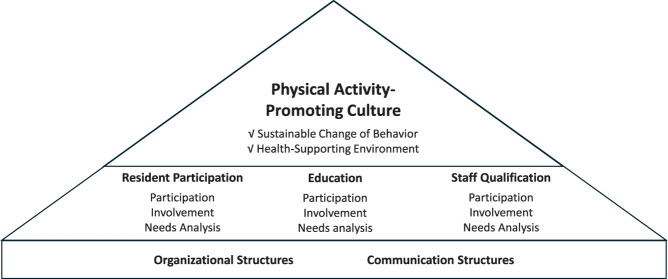
The PROGRESS pyramid.

The identified organizational and individual determinants were transferred into the PROGRESS pyramid (see Figure 2). This conceptual framework synthesizes the essential building blocks necessary for the sustainable implementation of a physical activity-promoting culture in nursing homes. The pyramid translates the study’s central findings into a structured model that highlights the hierarchical and interdependent nature of the elements necessary to integrate exercise and environmental interventions into nursing home practice.

At its foundation, the PROGRESS pyramid emphasizes the importance of organizational and communication structures, which emerged as indispensable prerequisites for implementation across all participating nursing homes. Participation opportunities, resident engagement strategies, education of all relevant groups, and staff qualifications are required from subsequent layers to enable consistent and effective delivery of interventions. Therefore, the PROGRESS pyramid serves as a practical, theoretically grounded tool for understanding how project-based initiatives can evolve into enduring nursing home practices.

The following sections elaborate on each dimension of the PROGRESS pyramid and describe how organizational and individual factors interact to facilitate or hinder the development of a sustainable physical activity–promoting culture in nursing homes.

### 4.2 Sustainable promotion of physical activity requires organizational structures

According to the setting-approach, the nursing home is a complex living environment in which structural, organizational, social, and individual factors influence health. To be effective in the long term, interventions must consider not only physical and psychosocial conditions, but also institutional structures, human resources, the needs of residents, and participatory decision-making processes (World Health Organization, Regional Office for Europe, 1986; Blüher and Kuhlmey, 2019). The requirements for the steering committee and needs assessment set out in the prevention guidelines are fundamental structures that pose a major challenge for nursing homes. As reflected in the workshop feedback and implementation patterns observed across facilities, coordination usually takes place at the team level (specific to professional groups and/or wards), without the results being exchanged between teams or structurally anchored. In addition, the attitudes of individual employees sometimes do not correspond to those of the team or management, and thus, measures to promote physical activity are implemented, communicated, and practiced on an individual basis, rather than forming part of a clear company-wide structure (Fleischmann et al., 2019).

Organizational structures also play a crucial role in shaping how physical activity opportunities are perceived and recognized, and in sustaining them over time. The present findings suggest that the way in which physical activity opportunities were embedded and communicated influenced whether residents perceived and recognized these opportunities as part of everyday life in the nursing home. For sustainable promotion of physical activity, opportunities to this end must be strategically anchored and prioritized at all hierarchical levels. This can and must be ensured by agreeing on activity measures in the steering group. Otherwise, preventive measures are often the first to be cut in the event of staff shortages or financial constraints (Marent et al., 2014; Dierich et al., 2019). Conducting regular needs assessments enables ineffective measures to be replaced and new strategies to be tested. This approach is particularly important given that engagement with physical activity opportunities was consistently lower than participation in the exercise program. Across environmental components, self-reported non-participation ranged from around 20%–45%, whereas non-participation in the exercise intervention was markedly lower (16.1%). These disparities highlight the need for ongoing monitoring to adjust strategies accordingly. For example, given that lack of awareness emerged as the most frequently reported barrier, improved promotion and communication strategies should be prioritized to increase participation in underutilized physical activity opportunities.

Despite implementation challenges, 86.0% of residents wanted the interventions to continue and 64.0% reported personal benefits. This acceptance underscores the importance of establishing long-term organizational structures. Once these are in place, a clear communication structure is required to disseminate needs assessment results and planned measures both internally and externally.

### 4.3 Sustainable promotion of physical activity requires communication and cultural change

In addition to the organizational structure, effective communication between the various professional groups within a nursing home (beyond the steering committee) is fundamental to the sustainable promotion of physical activity. A lack of transparency and the insufficient involvement of nursing staff have been identified as key obstacles to implementing measures to promote physical activity (Marent et al., 2014; Horn et al., 2019). If, for example, the steering committee becomes active within the framework of PROGRESS to identify needs and develop measures, the entire process (from needs assessment to implementation) must be clearly communicated and comprehensible to all parties involved (employees, residents, relatives, therapists, doctors, etc.), both internally and externally (Bender et al., 2018; Horn et al., 2019).

The steering committee’s task encompasses two key areas of communication: internal communication within the nursing home (with employees and residents) and external communication with external parties (relatives, doctors, therapists, etc.). During the PROGRESS study, various issues with internal and external communication emerged: (1) Although information was discussed within the steering committee, it was not communicated internally and therefore did not reach employees and residents in the nursing home. This lack of information transfer means that information events are not well attended, and the nursing home has difficulty implementing new measures. These communication gaps were reflected in our participation data. The vast majority (90.4%) of residents engaged in at least one guided intervention, but many reported being unaware of program offerings, not receiving information, or forgetting about opportunities. These barriers could have been avoided with more effective communication strategies. (2) External communication with relatives, doctors, or therapists was also challenging. On the one hand, there was often skepticism within nursing homes about external communication. On the other hand, important information was frequently lost when internal decisions were communicated to external parties.

Beyond general awareness issues, the feedback interviews revealed that communication gaps had created specific behavioral barriers. When asked why they did not use environmental interventions, residents cited “not knowing if they were allowed” or “not realizing they were included”, despite these physical activity opportunities having been developed specifically for them. This was problematic, given that 63.0% of residents never used environmental interventions outside of group sessions, despite the necessary facilities and equipment being available. Moreover, of those who used the outdoor physical activity opportunities, 19.2% only did so when accompanied by staff, and none of the residents engaged with them independently. This highlights how dependent physical activity opportunities are on active staff involvement rather than functioning as self-initiated opportunities. These findings demonstrate that unclear communication about the physical activity-promoting culture can promote residents from understanding their options and feeling empowered to participate, even after some structural barriers have been eliminated. Therefore, sustainable implementation depends not only on initial communication, but also on repeatedly reinforcing the purpose, availability, and accessibility of the physical activity opportunities.

The early and active involvement of employees from different functional groups, beyond the steering committee, increases acceptance and improves the willingness of individual stakeholders to embrace change. Horn et al. (2019) demonstrated that, while employees are often skeptical and/or resistant at first, this decreases as soon as the positive effects of health promotion measures on residents’ physical, cognitive and psychosocial wellbeing become apparent. Huber (2024) also emphasize the role of managers, whose active involvement and support can promote cultural change and reduce resistance. Studies also show that interprofessional collaboration, continuous training and supportive structures improve communication and promote successful long-term implementation (Dierich et al., 2019; Krupp et al., 2021). However, effective employee engagement requires addressing practical concerns. For example, staff expressed skepticism about planning four sessions per week, fearing that this frequency could discourage residents from participating. This feedback demonstrates the importance of two-way communication, allowing employees to voice concerns about implementation and informing them of the actual program.

Another key aspect of communication is engaging with residents and their relatives. While residents’ needs and wishes regarding mobility are highly individual, they are often not adequately recorded or communicated. Consequently, both residents and staff may underestimate their abilities (Drubig et al., 2023). Studies indicate that communication strategies which actively involve residents and their family members positively affect participation in physical activity programs (e.g., through information events or joint decision-making processes) (Dechamps et al., 2010; Vseteckova et al., 2018; Wiśniowska-Szurlej et al., 2020; Naqvi et al., 2025). As previously mentioned, the findings from the feedback interviews highlight critical gaps in resident communication. Analysis of reasons for non-participation revealed that issues of awareness dominated. Consistent with this, analysis of open-ended reasons for non-participation revealed that a lack of awareness and motivational factors were the most frequently cited barriers across all components. By contrast, purely health-related limitations (e.g., the use of a wheelchair, dizziness, and visual impairment) accounted for only a small proportion of responses. These communication failures directly impacted engagement. Environmental interventions had notably lower participation rates than guided exercise sessions. For example, 44.4% of residents self-reported never using the indoor walking brochures, whereas only 16.1% self-reported never attending exercise sessions. This is partly because residents lacked the ongoing reminders and guidance that made exercise sessions more salient. Furthermore, clear, bidirectional communication enables programs to be adapted to the residents’ living environment, which in turn promotes acceptance and participation (Marent et al., 2014; Horn et al., 2019). Another study showed that implicit communication of social norms, for example, via newsletters and images on physical activity in old age, significantly increases self-reported participation in physical activity programs (Rayling et al., 2024). This illustrates how subtle forms of communication can strongly influence behavior, and thus cultural change. Our findings also revealed that extrinsic motivators, such as certificates and stamp cards, which can be viewed as ongoing communication touchpoints, are vital elements for encouraging participation. This highlights the importance of sustained, repeated communication.

Family engagement presented challenges. Only 5.5% of residents took advantage of the opportunities to be physically active with their relatives. This suggests that external communication strategies need to be improved to encourage family members to support and reinforce participation.

Due to the multifaceted communication challenges identified in literature and in our study, specially trained physical activity ambassadors and assistants play a key role in the PROGRESS concept. They act as a cross-sector communication interface, addressing the communication gaps revealed in our feedback interviews. They facilitate communication across residents, relatives, colleagues, nursing staff, management, other external specialists, and the steering committee, thereby addressing the communication gaps identified in the feedback interviews. In the PROGRESS concept, ambassadors will be trained to provide consistent reminders, guidance, and motivation, rather than relying on information that often fails to reach residents through existing channels. Residents identified these as vital to engagement. They also actively promote a culture of physical activity within the nursing home. For this to succeed, the nursing home must consciously decide to become a physical activity-promoting organization and clearly communicate this both internally and externally. This organizational decision is essential because our findings demonstrated that individual measures alone cannot overcome systemic communication barriers. Introducing and implementing physical activity-promoting measures requires additional structural adjustments and a conscious cultural change that must be supported by everyone involved. The positive findings that 86.0% of participants want the interventions to continue, and that 64.0% have experienced benefits demonstrate residents’ receptiveness to such a cultural change when it is communicated and supported properly. Physical activity promotion can only be successfully established in the long term if the additional effort is transparent and shared (Wollesen et al., 2023).

### 4.4 Sustainable promotion of physical activity requires participation of residents

Participation in promoting physical activity as a dimension of health promotion can be implemented at various levels. A recent study involving seven nursing homes shows that participatory approaches can successfully promote physical activity in three areas: activities of daily living (e.g., setting the table, folding laundry or baking); structured, guided activities (e.g., walks); and the design of an activity-promoting environment (Hahn et al., 2024).

Older adults often experience a loss of autonomy when moving into a nursing home, which can directly impact their willingness to engage in physical activity (Lord et al., 2016; Scheibl et al., 2019). Often, doctors or family members make decisions about placement instead of the individuals themselves, limiting their autonomy from the outset (Johnson et al., 1994).

Several studies report that fixed organizational structures and routines in facilities, control by staff, and rigid guidelines with inflexible procedures leave residents with little opportunity to participate and do not meet their needs (Ryan and Scullion, 2000; Davies and Nolan, 2003; Davies and Nolan, 2004; Graneheim et al., 2013; Afram et al., 2014; Seiger Cronfalk et al., 2017; Scheibl et al., 2019). Standardized schedules can reinforce the perceived dependence and reduce the intrinsic motivation for physical activity. As a result, residents often exhibit selective participation, resignation or stoicism, accepting limited independence (Langer and Rodin, 1976; Johnson et al., 1994; Brooker, 2008; Heid et al., 2014). However, studies show that even modest choices can improve wellbeing and the willingness to be active (Langer and Rodin, 1976).

Although the literature emphasizes autonomous decision-making and self-direction, our feedback interviews revealed a more nuanced understanding of what it means to be actively involved for nursing home residents. Rather than seeking complete autonomy, residents expressed a need for what could be termed “supported participation”, involvement in activities with the right amount of guidance, structure, and encouragement. This may differ between new residents and those who have stayed for a longer period of time. Qualitative analysis revealed that guidance and reminders are essential for participation, as are extrinsic motivators, such as certificates and stamp cards. These findings challenge the assumption that external motivation undermines intrinsic motivation in this population. Rather, they suggest that many residents require and value external support to maintain participation. While 90.4% of participants engaged in at least one guided intervention, 37.0% took advantage of physical activity opportunities outside of structured group sessions, despite the necessary equipment and spaces being freely available. This difference in participation rates suggests that opportunity alone does not constitute meaningful access for many nursing home residents without guidance and structure.

However, promoting physical activity through true participation means more than just choosing from a selection of pre-determined activities. It involves residents at every stage, from analyzing needs and planning action strategies to evaluation. Furthermore, participatory physical activity promotion increases physical activity and can improve self-esteem and quality of life. A study of nursing home residents exhibiting depressive symptoms revealed that an intervention based on self-determination theory reduced depressive symptoms and helped maintain physical activity levels (Chen and Li, 2014). This leads to the idea that when residents can decide how, when and with whom they are active or exercise, their willingness to participate and long-term motivation increases. However, our findings suggest that the way residents decide “how, when, and with whom” may differ from what was anticipated. They may prefer guided participation to independent activity. They may also prefer extrinsic rewards that provide structure and recognition. Furthermore, they may value the social and supportive elements of structured sessions over the flexibility of self-directed physical activity opportunities. This could also arise from the fact that these kinds of activities and autonomous behavior were newly introduced to the residents, and they were not used to them and could not adapt during the time of the project. Residents may be more willing to participate in physical activity programs if they are involved in planning and decision-making processes (Hahn et al., 2024).

In summary, sustainable participation and involvement require an ongoing process of engaging with residents, including the evaluation and adaptation of activities. Qualitative research with nursing home residents shows that their mobility-related needs and wishes are highly individual but rarely expressed to nursing staff (Drubig et al., 2023). Our feedback interviews confirmed this pattern. Analysis of reasons for non-participation revealed diverse individual barriers, including health limitations (e.g., reduced mobility, visual impairments, and dizziness), personal preferences (e.g., preference for outdoor activities and following one’s own routines), and social factors (e.g., not wishing to engage with other participants). This heterogeneity highlights why standardized approaches cannot meet all residents’ needs. Therefore, regular feedback loops between residents and the care team are just as important as systematically documenting and considering individual preferences. Only by continuously comparing physical activity-promoting services with individual physical activity-related motives and goals can ensure that physical activity promotion meets residents’ needs and wishes (Hahn et al., 2024).

### 4.5 Sustainable promotion of physical activity requires education

To successfully and constructively involve all stakeholders, they must be educated and informed about the relevance and positive aspects of physical activity and exercise (Schaller et al., 2024). As the body reacts with a steady, long-term decline in motor and cognitive abilities when no new stimuli are received, it is important to educate all stakeholders about the need for daily physical activity and cognitive stimulation (Volkers and Scherder, 2011; Leon and Woo, 2018).

To date, nursing homes often lack a clear understanding of how to promote physical activity effectively. The way in which this is implemented varies greatly and is often unsystematic (Hasseler, 2011; Horn et al., 2011; Kuhlmey, 2012; Vanheiden, 2017). Although many care theories address the promotion of physical activity, this focus is not consistently reflected in nursing home practice. Instead, care provision tends to prioritize residents’ limitations, such as mobility, cognition, or mental health, rather than their remaining resources and opportunities for participation. This indicates a continued emphasis on a deficit-based approach, which prioritizes identifying and compensating for existing limitations rather than promoting existing resources (Wingenfeld et al., 2002; Bartholomeyczik et al., 2008; Hasseler, 2011; Vanheiden, 2017; Blättner et al., 2018).

In the context of health promotion, measures to promote physical activity should be part of a comprehensive, participatory, setting-related and resource-oriented concept. These measures differ from traditional, deficit-oriented care, which focuses on compensating individual limitations through isolated nursing actions rather than systematically promoting existing resources. In practice, however, the boundaries between these two concepts become blurred, as nursing staff view both approaches as interrelated aspects of their work. Nevertheless, it is crucial to distinguish between measures that promote physical activity and activating care, as this necessitates a shift in mindset from deficit-oriented to resource-oriented thinking, which is essential for contemporary health promotion (Fleischmann et al., 2019).

The fact that 85.1% of residents did not notice staff wearing pedometers and only 5.6% found them motivating indicates that the intended role-modelling function of staff was not realized. At the same time, 95.7% of residents who wore a pedometer themselves considered the wearing time appropriate, suggesting that, with better education and visibility, pedometers could have been a more powerful tool for both residents and staff. This suggests a lack of knowledge regarding educational and sports science approaches. Furthermore, the poor results in terms of offerings for relatives or staff alone indicate an urgent need for education. Everyone involved needs to understand why physical activity is so important, and this education is necessary until all stakeholders can provide more positive arguments than excuses or potential counterarguments regarding physical activity.

Relatives can contribute to this by encouraging and motivating their relatives to participate in suitable events at the nursing home. For example, during the PROGRESS project, many residents were discouraged from participating by their relatives. During the PROGRESS project, many residents were discouraged from participating by their relatives. The reason given was usually: “It is too much for my mother/father” (quote from an informational discussion within the PROGRESS study). Previous studies also show that relatives are sometimes skeptical about measures to promote physical activity in nursing homes (Janßen, 2019). Not only relatives, but also medical staff was sometimes reluctant to participate in interventions promoting physical activity, with the result that some residents refused to take part in the study on medical advice or withdrew their participation after initially showing interest and consulting a doctor. Information events and flyers can help to reduce skepticism among relatives and doctors regarding physical activity promoting measures (Josat, 2005; Bender et al., 2018; Horn et al., 2019; Janßen, 2019; Drubig et al., 2023). This creates a participatory process of raising awareness and rethinking.

### 4.6 Sustainable promotion of physical activity requires qualification

Depending on whether the programs are carried out by internal staff or external trainers, promoting exercise in nursing homes presents various challenges and advantages (Lazowski et al., 1999; Maritz, 2007; Brach et al., 2009; Jansen et al., 2014; Shakeel et al., 2015). Some studies suggest that a shortage of human resources, knowledge and training makes it challenging for nursing home staff to independently and consistently implement exercise programs (Brach et al., 2009; Kuk et al., 2018; Frahsa et al., 2020; Hirt et al., 2024; Trollebø et al., 2024). The shortage of staff and the economic situation in German nursing homes pose an obstacle to preventive services and, in many cases, threaten their very existence (Statistisches Bundesamt (Destatis), 2024). It is hardly feasible for nursing homes to permanently employ and pay qualified trainers at market rates from their own resources. However, the professional and effective implementation of group-based physical exercise intervention programs that include progression and individual adjustments requires the expertise of a qualified trainer in sport science. This creates a conflict between financial possibilities and quality standards that need to be resolved. Several studies report that, while the initial use of qualified external trainers can be helpful, it creates long-term dependencies and programs often lose momentum without internal training (Brach et al., 2009; Heid et al., 2014). To prevent prevention programs from becoming ineffective after external research or pilot projects are completed, and to meet the sustainability and personal responsibility requirements of the prevention guidelines, employees must be systematically trained on site (Levinger et al., 2023; Wollesen et al., 2023; Schaller et al., 2024). Without such training, nursing homes become permanently dependent on external services and funding projects.

One possible solution are structured and practical training measures that provide employees with the necessary knowledge and skills to promote physical activity within the nursing home’s daily routine. Participatory training concepts involving practical exercises, mentoring and ongoing support have proven particularly effective (Brach et al., 2009; Kuk et al., 2018; Vikström et al., 2021; Trollebø et al., 2024). Close cooperation between nursing staff and external experts also ensures acceptance and long-term implementation (Lazowski et al., 1999; Jansen et al., 2014; Hirt et al., 2024; Trollebø et al., 2024). In addition to cost efficiency, sustainability is a decisive factor when comparing internally and externally managed programs. Well-trained internal teams are therefore crucial for sustainable implementation. External offerings should primarily be a transitional solution (Lazowski et al., 1999; Maritz, 2007; Brach et al., 2009; Jansen et al., 2014; Frahsa et al., 2020; Vikström et al., 2021).

Informal discussions with the employees revealed that staff members clearly recognized the importance of adequate training for the long-term sustainability of physical activity programs. Several reported that they felt unprepared or overwhelmed by the prospect of leading group-based physical exercise sessions, citing limited experience and insufficient pedagogical or movement-related skills. These concerns were exacerbated by broader financial and staffing constraints within the nursing homes, which the staff perceived as limiting their ability to support such responsibilities. While some staff members were willing to continue the activities, they emphasized that protected time and explicit support from their employers would be essential for sustained implementation.

When course programs are not led by experts, it is often necessary to work at a lower intensity level. This ensures the safety of participants and meets the qualification requirements for course instructors. Thus, the low threshold approach represents a possible compromise, enabling individuals with prior knowledge of health sciences to qualify to lead such programs through appropriate training, although the capacity to deliver adequately dosed and progressive training stimuli may be limited.

However, the qualification not only refers to the implementation of group programs (exercise interventions) but also includes designing the living environment to promote physical activity (environmental interventions). Training is also required to design interior and exterior areas of nursing homes to promote physical activity. The key challenge when developing environmental prevention measures is translating the mostly abstract needs of residents into concrete, everyday activities for physical activity, for example, with low-threshold physical activity opportunities (activity posters or ball games) or signposted walks. The aim is to use or adapt architectural and spatial conditions in such a way that they promote everyday physical activity without requiring additional programs. The qualification therefore teaches skills for analyzing spatial potential, developing participatory interventions for increasing physical activity, and implementing them in practice, considering safety aspects, aesthetics, and the individual mobility restrictions of residents.

By training employees to become physical activity ambassadors and assistants, nursing homes can implement and carry out need-based and participatory physical activity programs independently and sustainably, even after the end of a study/funding period. This allows nursing homes to independently and sustainably continue exercise and environmental prevention measures, thus establishing physical activity as a permanent fixture in institutional culture.

### 4.7 A sustainable physical activity-promoting culture requires exercise and environmental prevention

A sustainable physical activity-promoting culture emerges when physical activity and its promotion are systematically integrated into all areas of the nursing home, from everyday care to the institutional mission statement. Staff and relatives need to set an example and actively promote a physically active lifestyle due to the decline in physical, cognitive, and psychosocial functions among multimorbid nursing home residents (Lazowski et al., 1999).

It is important to note that the “Health-Supporting Environment” at the top of the PROGRESS pyramid (see Figure 2) refers to the framework’s overall institutional goal rather than to a specific study component. As outlined in this section, a culture that promotes physical activity and is sustainable, constitutes a health-supporting environment in the sense of the setting-based approach to health promotion. In this approach, physical, organizational, social and communicational conditions work together to enable and sustain health-oriented behavior (World Health Organization, Regional Office for Europe, 1986; GKV-Spitzenverband, 2024).

The present findings suggest that group-based physical exercise and environmental interventions fulfil distinct but complementary functions within a culture that promotes sustainable physical activity. Group-based exercise interventions focus on physical components such as muscle strength, balance, gait stability, and cognitive functions, while simultaneously providing clearly structured, time-bound, and socially framed opportunities for participation, facilitating recognition, recall, and attribution of engagement (Belkin et al., 2025). These characteristics make exercise interventions particularly effective in supporting targeted improvements in physical functioning and fostering a shared understanding of participation among residents and staff.

In contrast, environmental interventions primarily operate by embedding opportunities for movement into everyday routines and the physical living environment. They are not as effective in discrete participation episodes as exercise interventions, but they have the potential to promote habitual physical activity and mobility throughout the day. Combining them allows the strengths of each approach to compensate for the limitations of the other. While exercise interventions enhance visibility, structure and motivation, environmental interventions increase reach and integrate physical activity into daily life. Together, they lay the foundation for a culture that promotes physical activity in nursing homes comprehensively and sustainably. Personal engagement and continuous motivation to participate in both forms of prevention are key.

As we know, there isn’t a one-size-fits-all solution. It is essential to offer nursing homes, and in turn, their residents, a range of options. The frequency of use and the quantitative and qualitative results of PROGRESS demonstrate the effectiveness of combining exercise and environmental interventions. Both forms of intervention were used and received positive feedback. Overall, 64.0% of participants reported personal benefits from the interventions.

Exercise affects not only motor and cognitive abilities, but also brings significant psychosocial and cognitive benefits that can lead to a noticeable improvement in quality of life (Langer and Rodin, 1976; Piedras-Jorge et al., 2010; Lok et al., 2017). This holistic effect means that promoting physical activity is a key part of improving quality of life in nursing homes. Incorporating the promotion of physical activity into the nursing home’s mission statement allows for an interdisciplinary or interprofessional approach to providing physical activity (Schaller et al., 2024). Only when physical activity is established as an institutional priority all professional groups can collaborate towards this goal. The success factors evaluated by PROGRESS can be applied to other concepts, forming the basis for the sustainable promotion of physical activity.

• Organizational structures: Clear responsibilities and processes within the institution.
• Communicational structures: Transparent and continuous coordination and a team-oriented approach.
• Participation: Active involvement of residents throughout the process.
• Education: Providing comprehensive information for all involved groups.
• Qualification: Continuous professional development of employees.

These five success factors build on each other, forming a pyramid. Together, they create conditions that promote an understanding of, and commitment to, physical functioning and physical activity as integral parts of care culture, rather than additional tasks.

## 5 Limitations

Several limitations should be considered when interpreting the findings. Firstly, participation in non-guided environmental intervention was not documented by nursing home staff, which limits the possibility of an objective assessment of independent use. This reflects routine practice in long-term care settings and highlights implementation challenges.

Secondly, the study was conducted in a limited number of nursing homes within one geographic region, which may restrict its generalizability. Nevertheless, the participatory design and diversity of intervention components increase the relevance of the findings for comparable long-term care settings.

Finally, the present article focused on implementation processes and determinants rather than clinical effectiveness outcomes. This deliberate focus aligns with the study’s aim to inform the sustainable integration of the promotion of physical activity into everyday nursing home practice.

## 6 Conclusion

The PROGRESS study shows that integrating group-based physical exercise programs with opportunities for physical activity can contribute to developing a culture that promotes physical activity in nursing homes when organizational structures and individual needs are addressed simultaneously. Developed from these findings, the PROGRESS Pyramid provides a structured framework that highlights essential building blocks for sustainable implementation, including organizational foundations, effective communication, resident participation, education, and staff qualifications. Coordinated implementation of these components may help nursing homes to shift from isolated, project-based activities to institutionalized practice. Further research is needed to provide practical recommendations and to examine how these building blocks can be adapted to different facility types and health systems to strengthen the long-term promotion of physical activity in institutional care settings.

## Data Availability

The raw data supporting the conclusions of this article will be made available by the authors, without undue reservation.
