# Supplementary material for: Promoting physical activity and physical functioning among residents in nursing homes – lessons learnt by the PROGRESS project
Source: Front Aging. 2026 Aug 3;7:1793208. doi: 10.3389/fragi.2026.1793208 (PMC13478141; doi:10.3389/fragi.2026.1793208)
Supplement: Supplementary file 1 [file Table1.docx]

Supplementary Table 1: Comparison of consistent and inconsistent recall about intervention attendance.

| Recall  Type of intervention | | Consistent recall | | | | Inconsistent recall | | | | | |
| --- | --- | --- | --- | --- | --- | --- | --- | --- | --- | --- | --- |
|  |  | TOTAL consistent | Never | Monthly | Weekly | TOTAL inconsistent | Never | Monthly | Weekly | No memory |  |
| Exercise intervention *n = 62* | | 49 (71.0%) | 7 | - | 42 | 13 (29.0%) | 3 | 1 | - | 4_a_  5_b_ |  |
| Guided environmental intervention *n = 65* | Activity posters *n = 46* | 23 (50.0%) | 9 | 7 | 7 | 23 (50.0%) | 9 | 2 | 3 | 8_a_  1_b_ |  |
|  | Seated bike *n = 65* | 40 (61.5%) | 16 | 18 | 6 | 25 (38.5%) | 8 | 7 | 3 | 3_a_  4_b_ |  |
|  | Darts *n = 30* | 9 (30.0%) | 2 | 7 | - | 21 (70.0%) | 8 | - | - | 11_a_  2_b_ |  |
|  | Ball games *n = 30* | 12 (40.0%) | 2 | 7 | 3 | 18 (60.0%) | 4 | - | 1 | 12_a_  1_b_ |  |
|  | Rollator club *n = 65* | 29 (44.6%) | 15 | 6 | 8 | 36 (55.4%) | 15 | 2 | 2 | 14_a_  3_b_ |  |
|  | Indoor walking brochures *n = 35* | 16 (45.7%) | 14 | 2 | - | 19 (54.3%) | 10 | 2 | 1 | 5_a_  1_b_ |  |

*Note*: a = participation documented by a trainer, b = no participation documented by a trainer. Values represent the number of participants (*n*) reporting each barrier. Dashes indicate that no participants reported that specific barrier for the given intervention.

Example of how to read the table: Of the 46 participants who received the exercise posters, 50% correctly remembered using them, while the other 50% did not. Of those with consistent recall, nine correctly answered that they had never used the posters; seven correctly answered that they had used them monthly; and seven correctly answered that they had used them weekly. Of those with inconsistent recall, nine said they had never used the posters, despite a trainer documenting their use. Furthermore, two said they used the posters monthly, and three said they used them weekly, but there was no record of any guided use. Of the nine participants who said they had no memory of using the posters, eight took part in guided use (a), and one did not (b).
