# Supplementary material for: Promoting physical activity and physical functioning among residents in nursing homes – lessons learnt by the PROGRESS project
Source: Front Aging. 2026 Aug 3;7:1793208. doi: 10.3389/fragi.2026.1793208 (PMC13478141; doi:10.3389/fragi.2026.1793208)
Supplement: Supplementary file 2 [file Table2.docx]

Supplementary Table 2: Barriers to independent participation.

| **Barrier category** | **Activity posters** | **Seated bike** | **Darts** | **Ball games** | **Rollator club** | **Indoor walking brochures** |
| --- | --- | --- | --- | --- | --- | --- |
| **Lack of awareness** |  |  |  |  |  |  |
| Not aware of the opportunity | 2 | 9 | 1 | 2 | 8 | 2 |
| Not invited | 1 | 7 | 4 | - | 3 | 4 |
| **Motivational factors** |  |  |  |  |  |  |
| Lack of interest | 3 | 1 | 1 | 2 | 2 | 4 |
| Feeling "too lazy" | 3 | — | — | — | 4 | 3 |
| Not engaging when passing by | 2 | — | — | — | — | — |
| Perceived as unhelpful | 1 | — | — | — | — | — |
| Explicit disinterest in activity | — | — | 4 | — | — | — |
| **Need for assistance** |  |  |  |  |  |  |
| lack of willingness to exercise alone | 2 | — | — | — | — | 4 |
| Functional limitations requiring assistance | — | 2 | — | — | 4 | — |
| Safety concerns | — | 1 | — | — | 1 | — |
| Had not received necessary accompaniment | — | — | — | — | 1 | — |
| **Individual constraints** |  |  |  |  |  |  |
| Frequent hospital stays | — | — | — | — | — | 1 |
| Preference for own routine | 2 | 1 | — | — | 3 | 4 |
| Use of personal equipment | — | 3 | — | — | — | — |
| Competing commitments | 1 | — | — | — | 2 | — |
| "Walked enough in life." | 1 | — | — | — | 1 | 2 |
| Not having thought about joining | — | — | — | 2 | — | — |
| Not seeing the point | — | — | — | — | 1 | — |
| All he/ she needs to know is that he/ she can still do the exercises | 1 | — | — | — | — | — |

*Note.* Values represent the number of participants (*n*) reporting each barrier. Dashes indicate that no participants reported that specific barrier for the given intervention.
